# Functional integration of services during the antenatal period can potentially improve childhood growth parameters beyond infancy: findings from a post-interventional follow-up study in West Bengal, India

**DOI:** 10.1186/s40795-024-00918-x

**Published:** 2024-08-15

**Authors:** Kayur Mehta, Sreeparna Ghosh Mukherjee, Ipsita Bhattacharjee, Kassandra Fate, Shivani Kachwaha, Tushara Rajeev, Aastha Kant, Meghendra Banerjee, Anita Shet

**Affiliations:** 1 Maternal and Child Health Center India, Johns Hopkins Bloomberg School of Public Health, Baltimore, MD USA; 2 International Vaccine Access Center, Johns Hopkins Bloomberg School of Public Health, Baltimore, MD USA; 3 Child in Need Institute, Pailan, West Bengal India; 4 Department of Health Policy and Management, Johns Hopkins Bloomberg School of Public Health, Baltimore, MD USA; 5 Department of International Health, Johns Hopkins Bloomberg School of Public Health, Baltimore, MD USA

**Keywords:** Maternal nutrition, Child undernutrition, Antenatal care, Functional integration, India

## Abstract

**Background:**

Despite progress, the prevalence of childhood undernutrition in India remains amongst the highest globally.

**Objective:**

We aimed to evaluate the impact of a functional integration interventional package during the antenatal period on childhood growth parameters.

**Methods:**

This is a post-interventional follow-up study of a maternal nutrition interventional study conducted between 2018 and 2019 among women in their first trimester of pregnancy from three districts in West Bengal, India. Pregnant women received a package of augmented interventions from study staff which supplemented those provided to them under the state-run programmes, that included body-mass-index measurement at pregnancy registration, monthly weight monitoring, targeted dietary counselling, supervised supplementary nutrition intake and iron-folic acid supplementation during daily anganwadi center visits. In the current follow-up study conducted in 2021, age-matched pregnant women from the same areas who were pregnant during the same period as in the original study and had received standard-of-care under the state-run programmes were recruited into a comparison group. Study staff collected data regarding maternal height and serial weights that were recorded at antenatal visits in 2018-19, and birth and infant characteristics. Child height and weight were measured during the follow-up visit in 2021, which were used to calculate the relative risks of stunting, wasting and underweight using generalized linear models, to understand the sustained impact of the intervention beyond infancy. Eight-hundred-nine mother-child dyads (406 intervention; 403 comparison) were followed.

**Results:**

Median age of women in the intervention and comparison group was 23 (IQR 20–25) and 25 (IQR 24–27) years respectively. Median gestational-weight-gain was higher amongst intervention group women (9 vs. 8 kg, *p* = 0.04). Low-birth-weight prevalence was 29.3% (119/406) and 32.0% (129/403) in the intervention and comparison group. At 12–35 months of age, children born to women in the intervention group had significantly reduced risk of stunting (RR = 0.65, 95% CI 0.44–0.94), wasting (RR = 0.57, 95% CI 0.33–0.97) and underweight (RR = 0.61, 95% CI 0.42–0.88).

**Conclusions:**

These results indicate that functional integration and strengthening of routine antenatal care services including targeted nutritional counselling to expectant mothers can have distal beneficial effects on childhood undernutrition beyond the immediate post-natal period.

**Supplementary Information:**

The online version contains supplementary material available at 10.1186/s40795-024-00918-x.

## Background

India carries one of the largest global burdens of childhood undernutrition. As per National Family Health Survey-5 (NFHS-5) estimates (2019–2021), the fraction of children under the age of 5 years in India who are stunted is 35.5%, and wasted is 18.5%, numbers far from global targets. In the Indian state of West Bengal, according to the NFHS-5 (2019–2021) estimates, 34% children under the age of 5 years are stunted and 20.2% are wasted [1], proportions that have increased from the previous survey conducted five years earlier (32.5% and 20.3% respectively) (NFHS-4, 2015–2016) [2].

Several factors have been implicated in the development of childhood undernutrition, and these include maternal factors such as fertility, height, Body Mass Index (BMI), age, socio-economic status, underlying anemia, nutrition during pregnancy, parity, birth order, inter-pregnancy interval, and childhood factors such as dietary intake, micronutrient supplementation, breastfeeding, complementary feeding, dietary diversity, and childhood morbidities and infectious diseases [3]. It is widely recognized that the key ‘window of opportunity’ for reducing childhood undernutrition is the first 1000 days from conception until 2 years of age [4]. The antenatal period, in particular, is a crucial window for the expectant mother, since nutritional requirements are high during pregnancy to support fetal growth.

In India, antenatal services are delivered through 2 major national programs: (1) the Integrated Child Development Services (ICDS) managed by the Ministry of Women and Child Development [5]; and (2) the Reproductive, Maternal, Newborn, Child, and Adolescent Health (RMNCH + A) strategy of the National Health Mission managed by the Ministry of Health and Family Welfare [6] (Supplementary Table 1). Whereas food supplementation, growth monitoring interventions and basic antenatal care are delivered through the ICDS programme, micronutrient supplementation, deworming, health check-ups, and curative interventions are delivered through the National Health Mission [5, 6]. Despite wide reach across the country, there are concerns with respect to implementation processes of both these programmes; studies evaluating the programmes conducted in different Indian states including West Bengal have identified gaps in infrastructure, manpower, training, record-keeping and supply chain interruptions, particularly related to supplemental nutrition delivery [7–10]. These observations call for effective “functional integration” of these systems. The WHO defines as “the extent to which key support functions and activities such as financing, human resources, strategic planning, information management, marketing and quality assurance/improvement are coordinated across all system’s units” [11]. It involves shared policies and practices for support functions across partnerships between different actors within a system. These linked systems can support and coordinate policymakers (system integration), managers (organizational integration), professionals (professional integration) and patients (clinical integration) in their accountability and shared decision-making in (inter-sectorial) partnerships [11].

In this study, we describe the effect of an intervention that functionally integrated the delivery of antenatal services in India, as an extension of a nutritional intervention project for expectant mothers that was initiated in 2018-19 in selected districts of West Bengal that studied pregnancy and birth outcomes [12].

## Methods

### Antenatal nutritional intervention study (2018–2019)

The original maternal and child health and nutrition project was conducted in between 2018 and 2019 in selected blocks of three districts of the state of West Bengal: Nagrakata block from Jalpaiguri district; Suti-I block from Murshidabad district; and Falta block from South 24 Parganas district in West Bengal, India. A total of 485 women who were in their first trimester of pregnancy in May 2018 and May 2019 received the intervention, that comprised of augmented antenatal care that supplemented standard interventions contained within the national guidelines under the ICDS and RMNCH + A programmes [5, 6]. In the study area, it was anecdotally observed that despite the emphasis on obtaining maternal height and weight measurements during antenatal visits, oftentimes this was not done. While iron and folic acid tablets were provided, adherence to intake was not monitored, and high-risk pregnancies were not followed closely. Hence, as a part of the intervention in this study, the following augmented interventions were delivered by the study staff in conjunction with the various cadre of frontline community health workers including those with the ICDS and RMNCH + A programmes, enabling functional integration across a spectrum of providers and services: (i) ensuring baseline maternal BMI measurement and monthly weight monitoring, (ii) ensuring daily on-the-spot supplementary nutrition (calorie dense food distributed through the ICDS scheme) intake by expectant mothers in their second and third trimesters of their pregnancies at ICDS centers – this process was supervised by study staff who ensured that the food was consumed in their presence 6 days/week) (iii) ensuring the intake of antenatal iron and folic acid supplementation, and (iv) providing targeted dietary counselling encompassing caloric needs and diet plans at each fortnightly visit based on nutritional status (BMI) as well as joint counselling of family members to ensure support for the expectant mother and sharing of household work burden and (v) identification and follow up of pregnant women at “nutritional risk” (A pregnancy was considered ‘at nutritional risk’ when at least one of the following indicators were present: a) BMI (taken < 20 weeks gestation) identified woman as severely thin, thin, overweight or obese b) Age of pregnancy (below 20 and above 35 years); c) Body weight at the time of registration (40 kg or less); d) Height (less than 145 cm); e) Anemia (classified as severe anemia: Hemoglobin less than 7 g/dl, moderate anemia: 7–10.9 g/dl, using measurements obtained at the first antenatal visit); f) Inappropriate gestational weight gain (GWG) (< 1 kg /month or > 3 kg /month from second trimester onwards [13]. These interventions were strengthened by study staff by ensuring increased home contact amongst identified women to ensure timely uptake of antenatal services, hemoglobin testing and treatment of anemia, ensuring ultrasonography after the second trimester and ensuring a fourth antenatal visit in the ninth month of pregnancy (all included in the national guidelines, but not necessarily done for/ availed by all pregnant women under prevalent conditions). All these interventions listed above were monitored closely by study staff.

### Child follow-up study (2021): selection of intervention and comparison groups for analyses

For this child follow-up study, mother-child dyads from the districts included in the original study were contacted and followed up from May 2021 to October 2021. Of the 485 women in the original study that received the intervention, the number available for follow-up was 406; exclusions were due to migration (*n* = 34), lost to follow up (*n* = 21), abortion or miscarriage (*n* = 10), stillbirths (*n* = 7) and child deaths (*n* = 7). We thus included all these women who were available and their children in the intervention arm of this follow-up study.

For the comparison group, we recruited age-matched women from the same geographic areas as in the original Antenatal Nutritional Intervention Study (2018–2019) who were in their first trimester of pregnancy in May 2018 and May 2019 and did not receive the interventions (confirmed by maternal records), but received the standard of care and were available for follow-up between May 2021 and October 2021. The women who met these criteria were randomly selected (using computer-generated algorithms) to constitute the comparison group (*n* = 403), which was chosen at the time of prospective data collection for this study.

### Study procedures, follow-up and outcomes

In the current study, follow-up was conducted between May 2021 to October 2021, when the offspring of intervention and comparison women were between 12 and 35 months of age. Study staff visited the homes of these participants and collected demographic details during interviews, and data regarding pregnancy, birth and infant characteristics were abstracted from Maternal and Child Protection Cards. Demographic details including maternal age, parity, birth interval, BMI at first antenatal visit, baseline maternal hemoglobin status (obtained from hemoglobin measurements during the first antenatal visit), maternal education status, number of antenatal visits, gestational weight gain, maternal hemoglobin at the third antenatal visit and mode of delivery were collected. Child details including the gestational age at birth, birth weight, and breastfeeding practices were obtained. At the follow-up visits, standard Seca scales were used to measure weight, and Seca infantometers and stadiometers were used to measure the length of children < 2 years of age and height of children > 2 years, respectively. Study staff took single readings of these measurements and recorded them in case record forms.

Primary outcomes of interest were stunting, wasting and underweight, defined as length-for-age, weight-for-height, and weight-for-age <-2 standard deviations (SD) from the median of the WHO child growth standards among 12–35-month-old children of women in the intervention group and comparison groups.

### Statistical analysis

Descriptive analyses were performed to assess the distribution of variables between the intervention and comparison groups. Proportions and medians [with interquartile ranges (IQR)] are presented for baseline variables. Chi-square tests were used to compare categorical variables between intervention and comparison groups, and sex differences in undernutrition in the study population. Analyses for gestational weight gain utilized a cut-off of 7 kg, which is the average gestational weight gain for pregnant women in India [14]. Anthropometric measures together with the age and sex of the children were used to calculate the weight-for-age (underweight), height for-age (stunting) and weight-for-height (wasting) z-scores according to the WHO growth standards. Relative risk of stunting, wasting, and underweight in children were compared between intervention and comparison groups using multivariable Poisson regression analyses after adjusting for baseline characteristics like household income and maternal and infant characteristics like mother’s BMI, maternal age, parity, baseline hemoglobin, birth interval, gestational age, gestational weight gain, exclusive breastfeeding for 6 months, and birth weight after checking the data for associations. Multivariable Poisson regression analyses were conducted to identify the relative risks of stunting, wasting and underweight associated with pregnancy and newborn related factors in the intervention and comparison groups. In the multivariable Poisson regression model, we included a priori all those factors that could be modified because of the intervention model as individual predictors in separate models for the intervention and comparison groups. Model selection for all analyses was guided by using the Akaike Information Criteria, using backward selection. These models were adjusted for the number of antenatal visits, hemoglobin at the third antenatal visit, gestational weight gain, birth weight, breastfeeding within an hour of birth and exclusive breastfeeding up to 6 months of age. Analyses were performed using STATA version 17.

### Ethical considerations

Written informed consent was obtained from parents or caregivers at the time of child enrolment in the current study. Ethics approval was obtained from the Johns Hopkins Bloomberg School of Public Health Institutional Review Board (No. 18366) and the Institutional Ethics Committee at the Child in Need Institute, Kolkata, India (No. 01/2021-22).

## Results

### Maternal and newborn characteristics

#### Antenatal factors

A total of 809 women with living offspring (406 in the intervention group; 403 in the comparison group) were included in the study. 53% (217/406) women in the intervention group and 44.4% (179/403) women in the comparison group were primiparous. At the time of pregnancy registration, 26.1% (106/406) women in the intervention group and 31.9% (128/403) women in the comparison group were underweight. 90% of the women had received four or more antenatal checks, and over half of the women were noted to be anemic during their first antenatal check. Median gestational weight gain recorded was greater amongst women in the intervention group compared to those in the comparison group (9 kg vs. 8 kg, *p* = 0.04) (Table 1).

**Table 1:** Characteristics of mothers and children (*n* = 809) †

Variable	Intervention group (*n* = 406)	Comparison group (*n* = 403)	*p* value
**Maternal characteristics**			
Median Age (in years) (IQR)	23 (20–25)	25 (24–27)	
Age			*p* < 0.001
< 18 years	17 (4.2)	0 (0)	
18–25 years	288 (70.9)	215 (53.4)	
26–34 years	96 (23.7)	188 (46.6)	
≥ 35 years	5 (1.2)	0 (0)	
Parity			
≤ 2	354 (87.2)	361 (89.5)	*p* = 0.01
≥ 3	52 (12.8)	42 (10.5)	
Birth Interval ≤ 2 years	11 (2.7)	29 (7.2)	*p* = 0.03
BMI at pregnancy registration			
≤ 18.5 (underweight)	106 (26.1)	128 (31.9)	*p* = 0.23
18.51–24.99 (healthy)	244 (60.1)	217 (54.1)	
25.0–29.9 (overweight)	47 (11.6)	44 (10.9)	
≥ 30 (obese)	9 (2.2)	12 (3.0)	
Baseline hemoglobin status			
≥ 11 g/dl (normal)	187 (46.1)	176 (44.1)	*p* = 0.88
10–10.9 g/dl (mild anemia)	187 (46.1)	192 (48.1)	
7.9–9.9 g/dl (moderate anemia)	30 (7.3)	30 (7.5)	
< 7 g/dl (severe anemia)	2 (0.5)	1 (0.3)	
Missing	0 (0)	4 (1.0)	
Maternal hemoglobin status at 3rd antenatal visit			
≥ 11 g/dl (normal)	208 (55.0)	185 (49.7)	*p* = 0.01
10–10.9 g/dl (mild anemia)	159 (42.1)	151 (40.6)	
7.9–9.9 g/dl (moderate anemia)	11 (2.9)	35 (9.4)	
< 7 g/dl (severe anemia)	0 (0)	1 (0.3)	
Missing	28 (6.9)	31 (7.7)	
Education status			
No formal schooling	32 (7.8)	25 (6.2)	*p* < 0.001
Upto Class 10	243 (59.9)	213 (52.8)	
Class 10 and above	131 (32.3)	165 (41.0)	
Total number of ANC visits ≥ 4	341 (83.9)	388 (96.3)	*p* = 0.004
Gestational weight gain (in kilograms)			
Median (IQR)	9 (7–11)	8 (6–10)	*p* = 0.04
< 7 Kg	92 (23.3)	122 (30.6)	
7-8.9 Kg	95 (24.0)	97 (24.3)	
>9 Kg	208 (52.7)	180 (45.1)	
Missing	11 (2.7)	4 (1.0)	
Place of delivery			
Home	3 (0.7)	11 (2.7)	*p* = 0.29
Institution	403 (99.3)	392 (97.3)	
**Infant characteristics**			
Male	198 (48.8)	199 (49.0)	*p* = 0.86
Gestational age			
Preterm (≤ 36 weeks)	59 (14.5)	40 (9.9)	*p* = 0.22
Term (37–41 weeks)	322 (79.3)	339 (84.1)	
Post-term (42 weeks)	25 (6.2)	24 (6.0)	
Birth weight (in Kilograms)			
Median (IQR)	2.7 (2.5-3.0)	2.7 (2.5-3.0)	*p* = 0.54
Birth weight ≤ 2.5 Kg (Low birth weight)	119 (29.3)	129 (32.0)	*p* = 0.27
Breastfed within 1 h of birth	360 (88.7)	211 (52.4)	*p* < 0.001
Exclusively breastfed up to 6 months	322 (79.3)	229 (56.8)	*p* < 0.001
Age at the time of contact††			
12–23 months	241 (60.4)	227 (56.3)	*p* < 0.001
24–35 months	158 (39.6)	176 (43.7)	
Missing	7 (1.7)	0 (0)	
Prevalence of undernutrition			
Overall (12–35 months)	*n* = 406	*n* = 403	
Stunted	72 (17.8)	124 (30.8)	< 0.001
Wasted	32 (8.0)	79 (19.7)	< 0.001
Underweight	64 (16.0)	134 (33.2)	< 0.001
12–23 months	*n* = 241	*n* = 227	
Stunted	35 (14.5)	57 (25.1)	0.004
Wasted	18 (7.5)	49 (21.6)	< 0.001
Underweight	35 (14.1)	64 (28.2)	< 0.001
24–35 months	*n* = 196	*n* = 199	
Stunted	44 (22.5)	64 (32.2)	0.03
Wasted	14 (7.1)	42 (21.1)	< 0.001
Underweight	25 (19.2)	70 (39.8)	< 0.001

†Data are n (column %) unless otherwise indicated

†† Age at the time of contact signifies the child’s age when the anthropometric measurements were recorded by the study team

#### Newborn outcomes

Births in a healthcare facility were recorded for 98.2% (795/809) children; birth at full-term was recorded for 79.3% (322/406) children in the intervention group and 84.1% (339/403) of the children in the comparison group. Low birth weight prevalence was 29.3% (119/406) in the intervention group and 32.0% (129/403) in the comparison group (Table 1). Details about commencement of breastfeeding and proportion exclusively breastfed upto 6 months are described in Table 1.

### Child growth outcomes

**Fig. 1:**
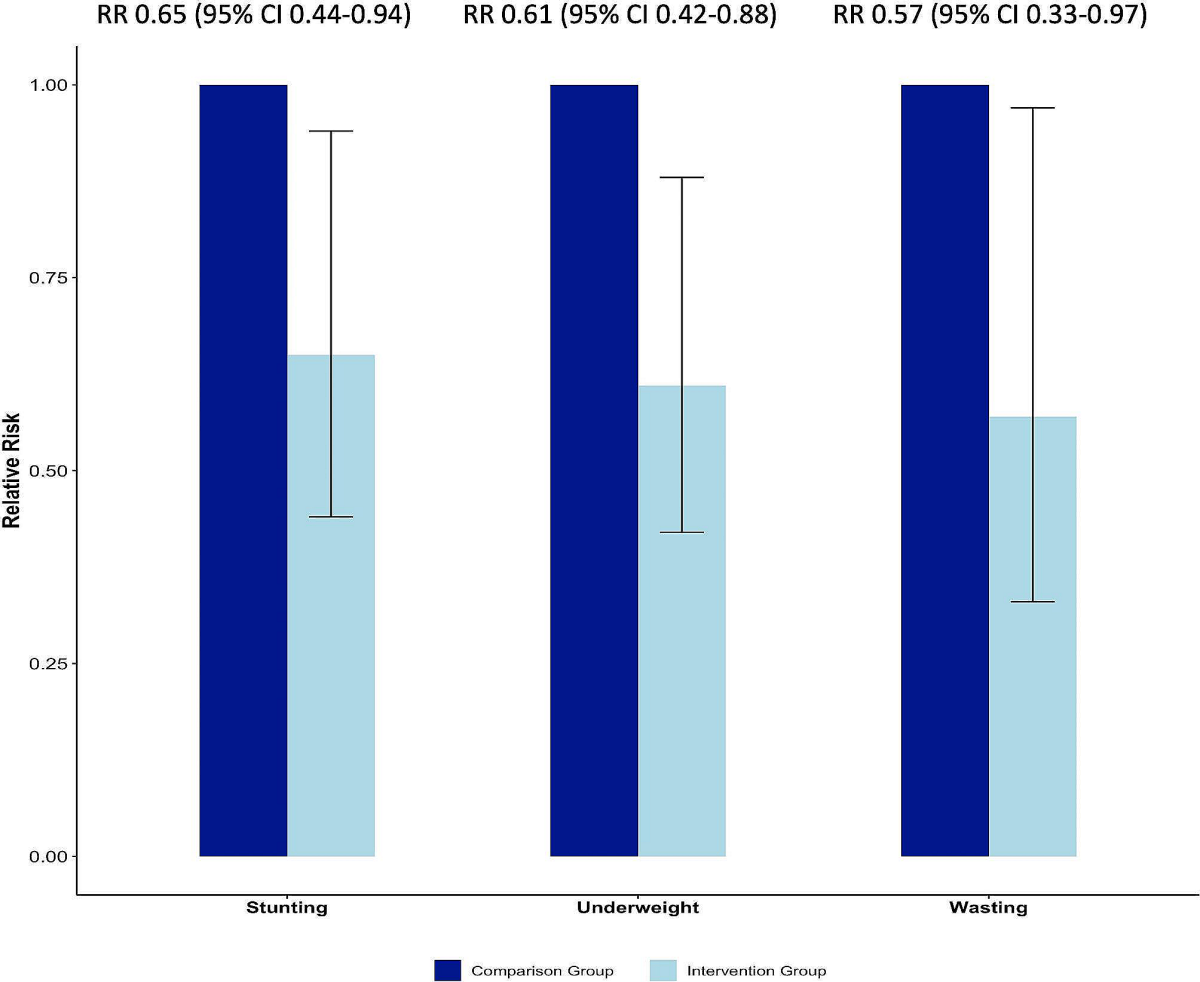
Association between the intervention and improved childhood growth indicators in children aged between 12–35 months. The relative risk of stunting, wasting and underweight in children aged between 12–35 months were significantly lower in the intervention group compared to the comparison group

The prevalence of stunting was 17.8% (72/399) and 30.8% (124/403) among children aged between 12 and 35 months in the intervention and comparison groups respectively (RR = 0.65, 95% CI 0.44–0.94). The prevalence of wasting amongst children in the intervention group was 8.0% (32/399) compared to 19.7% (79/401) of those in the comparison group (RR = 0.57, 95% CI 0.33–0.97). The prevalence of underweight amongst children in the intervention group was 16.0% (64/399) compared to 33.2% (134/403) those in the comparison group (RR = 0.61, 95% CI 0.42–0.88) (Fig. 1). The prevalence of undernutrition (stunting, wasting and underweight) was higher amongst children aged 24–35 months compared to those aged 12–23 months (Table 1). Overall, a greater proportion of males (108/395, 27.3%) were found to be stunted, compared to females (87/407, 21.4%) (*p* = 0.049); however no statistically significant sex differences were found for underweight and wasting (Supplementary Fig. 1).

### Pregnancy and newborn related factors associated with childhood undernutrition in the intervention and comparison groups

After adjusting for baseline demographic characteristics, we found that the relative risk of remaining stunted at 12–35 months for children born with low birth weight continued to remain significant amongst children in the comparison group (RR 1.61, 95% CI 1.14–2.24), but not amongst those in the intervention group (RR 1.17, 95% CI 0.63–2.04), suggesting beneficial effects of the intervention. Similar observations were noted for being underweight amongst children in the intervention and comparison groups (Table 2).

**Table 2:** Pregnancy and newborn related factors associated with undernutrition at 12–35 months among children in the intervention and comparison groups

Variable	RR of stunting		RR of wasting		RR of underweight	
	Intervention	Comparison	Intervention	Comparison	Intervention	Comparison
Number of antenatal visits </= 3	1.02 (0.52–1.85)	0.69 (0.22–1.63)	0.90 (0.27–2.35)	1.32 (0.37–3.42)	1.22 (0.61–2.25)	0.82 (0.31–1.74)
Low birth weight	1.17 (0.63–2.04)	1.61 (1.14–2.24)	2.57 (1.12–5.48)	1.87 (1.17–2.92)	1.69 (0.93–2.92)	1.70 (1.23–2.32)
Gestational weight gain < 7 Kg	1.84 (1.06–3.13)	1.66 (1.15–2.39)	1.11 (0.43–2.64)	0.81 (0.47–1.35)	1.71 (0.96–3.02)	1.66 (1.17–2.34)
Anemia at 3rd antenatal visit	1.23 (0.79–1.93)	1.14 (0.83–1.56)	0.87 (0.41–1.80)	1.65 (1.07–2.60)	1.14 (0.70–1.86)	1.31 (0.97–1.77)

## Discussion

The findings in this study indicated that a package of antenatal interventions involving functional integration approaches and targeted nutritional counselling during pregnancy were associated with improved child nutritional outcomes beyond immediate birth outcomes. The impact of the intervention that was delivered during pregnancy was demonstrable up to three years after cessation of the intervention, as children aged between 12 and 35 months born to women in the intervention group were less likely to be stunted, wasted or underweight in comparison to their counterparts in the non-intervention group.

Our intervention paid specific attention to nutritional counseling of the expectant women in the intervention group. The impact of nutrition education and counselling during pregnancy on birth weight and other pregnancy outcomes has been previously reported in earlier studies, and in a 2012 meta-analysis of 13 studies [15]. The meta-analysis noted that nutrition education and counselling was associated with an increase in mean birth weight (+ 105 g), but this was significant only when nutrition education was coupled with nutritional support in the form of food supplements, micronutrient supplements or nutrition safety net interventions [15]. The authors, however, did not report the impact of these interventions on birth length. While it is plausible that the improvements in health outcomes in these studies were secondary to improvements in dietary intakes and micronutrient supplementation, the role of nutrition counseling cannot be discounted. The WHO recommends nutritional counselling for all pregnant women [13]; however, this is often overlooked due to various reasons including lack of integration into primary health care services, poor access to healthcare, insufficient healthcare provider knowledge and lack of adaptation to contextual settings [16].The counselling provided to women in the intervention group in our study also could have had a beneficial effect on breastfeeding practices; much larger proportions of women in the intervention group commenced breastfeeding within 1 h of birth, and exclusively breastfed their children up to 6 months of age. In a study from Tanzania, researchers used a quasi-experimental evaluation design to evaluate the effectiveness of health and nutrition education during the 1000-day window, and found that the proportion of stunted children decreased from 35.9 to 34.2% in intervention sites, and there were increases noted in breast feeding within 1 h of birth [DID: 7.8%, (95% CI: 2.2–13.4), *p* = 0.006] and exclusive breast feeding in children under 6 months [DID:20.3%, (95% CI: 10.5–30.1), *p* = 0.001] [17]. Although we were not able to assess the postnatal nutritional practices amongst children in both groups in this study, it is likely that the nutritional counselling provided to women in the intervention group may have had an important impact in the subsequent child feeding practices practiced by the women in the intervention group, and may have contributed to the decreased risk of childhood undernutrition in the children born to these women. Our findings underscore the need to strengthen nutritional counselling services during antenatal care. Further, in the original study, and well as this follow-up study, we found that there was an increased gestational weight gain amongst mothers who received the intervention, amongst other improved maternal outcomes including reduction in the prevalence of anemia by the end of the pregnancy [12]. Findings from our study support the inclusion of gestational weight gain monitoring in routine antenatal care in India.

There has been a growing interest in functional integration as an approach to improve the implementation of existing interventions to achieve positive maternal and child nutrition outcomes. Functional integration is a term used within health systems strengthening circles and encompasses the coordination of key support functions such as strategic planning, human resources, financial management, information management and quality improvement to improve optimal health outcomes [18, 19]. In our study, the intervention saw the functional integration of various players in maternal and child health, such as Accredited Social Health Activists (ASHAs), Auxiliary Nurse and Midwives (ANMs) and Anganwadi workers (AWWs) working under different ministries namely health and family welfare, education, and women and child development under the Government of India, and civil society members or non-governmental organizations (NGOs) and academia. The intervention demonstrated the effectiveness of functional integration of antenatal care services in improving and having a long-lasting impact on childhood growth outcomes, and our findings advocate for scale-up of similar endeavors.

While our findings did not suggest immediate differences between intervention and comparison groups related to birth weight, an analysis of post-intervention factors associated with stunting showed a higher risk attributable to low birth weight in the comparison compared to the intervention group. This suggests potential sustained impacts of the intervention in the long-term, which could be explained by several biological mechanisms including better nutrition during pregnancy and better breastfeeding and child feeding practices. The impact of early-life nutrition interventions on long-term health outcomes has been well described, especially in the context of long-term cardiometabolic health [20]. However, very few studies have examined long-term impacts of interventions on childhood growth indicators; a recent study showed that India’s mid-day meal school feeding program was associated with intergenerational benefits in child linear growth [21]. Our study reports similar beneficial effects of the intervention manifesting up to three years after the intervention was stopped, suggesting that the antenatal intervention may have led to maternal behavior change post-partum that positively impacted child growth, despite being born with low birth weight, and thus these findings are of considerable public health significance.

The limitations of this study included a small sample size, lack of sample size calculations owing to the retrospective nature of this study, and a comparison group that was not systematically matched to the intervention group. We were unable to adjust for clustering at the district level during analyses, because of limitations associated with the study design. The follow up of the mother-child dyads in both groups was initiated after approximately two years after the antenatal intervention and was not done on a continuous basis. We did not have access to the maternal antenatal visit records as well as anthropometric records of the children at birth, especially length at birth, and hence are unable to report the extent of coverage of antenatal services and whether the children were stunted or wasted at birth. Anthropometric measurements were not taken twice to account for inter-observer variation. In addition, we did not have access to data on dietary intake of children, which is an important determinant for nutritional outcomes. The extent of functional integration was also not monitored. Lastly, the study was conducted in one state in India, limiting the generalizability of these findings. Despite these limitations, this is amongst the first studies to describe the effect of interventions given during pregnancy and their long-term impact in terms of childhood growth parameters 2–3 years after the cessation of the intervention, filling an important data gap, as most studies do not follow children longitudinally for such extended intervals of time.

In conclusion, our study points to the potential of strengthening and functionally integrating antenatal care as an effective approach to reduce the prevalence of childhood undernutrition, the potential impact of focused nutritional counselling to the expectant mother, and its longitudinal effects in terms of improved nutritional outcomes amongst young children. While larger studies that could overcome our potential limitations would be required to further strengthen this hypothesis, based on the promising findings of this study, we would recommend stronger action to advocate for and strengthen programmatic adherence to baseline maternal BMI measurements and monthly weight monitoring into routine antenatal care. We emphasize incorporating functional integration measures to ensure the effective uptake of maternal nutritional supplementation during the antenatal period, and providing targeted dietary counselling based on maternal nutritional status. These simple measures provided during the antenatal period could go a long way in not only improving maternal and birth outcomes, but also can have distal beneficial effects on childhood growth parameters beyond the immediate post-natal period.

### Electronic supplementary material

Below is the link to the electronic supplementary material.

**Supplementary Fig. 1**: Prevalence of undernutrition at 12–35 months by sex. The prevalence of stunting, wasting and underweight was higher amongst males as compared to females, and was statistically significant for stunting

Supplementary Material 2

## Data Availability

The datasets used and/or analysed during the current study are available from the corresponding author on reasonable request.

## Abbreviations

ANM: Auxiliary Nurse and Midwife
ASHA: Accredited Social Health Activist
AWW: Anganwadi Worker
BMI: Body Mass Index
ICDS: Integrated Child Development Services
NFHS: National Family Health Survey
RMNCH + A: Reproductive, Maternal, Newborn, Child, and Adolescent Health
WHO: World Health Organization
