# Supplementary figures and images for: Functional integration of services during the antenatal period can potentially improve childhood growth parameters beyond infancy: findings from a post-interventional follow-up study in West Bengal, India
Source: BMC Nutr. 2024 Aug 15;10:112. doi: 10.1186/s40795-024-00918-x (PMC11325605; doi:10.1186/s40795-024-00918-x)

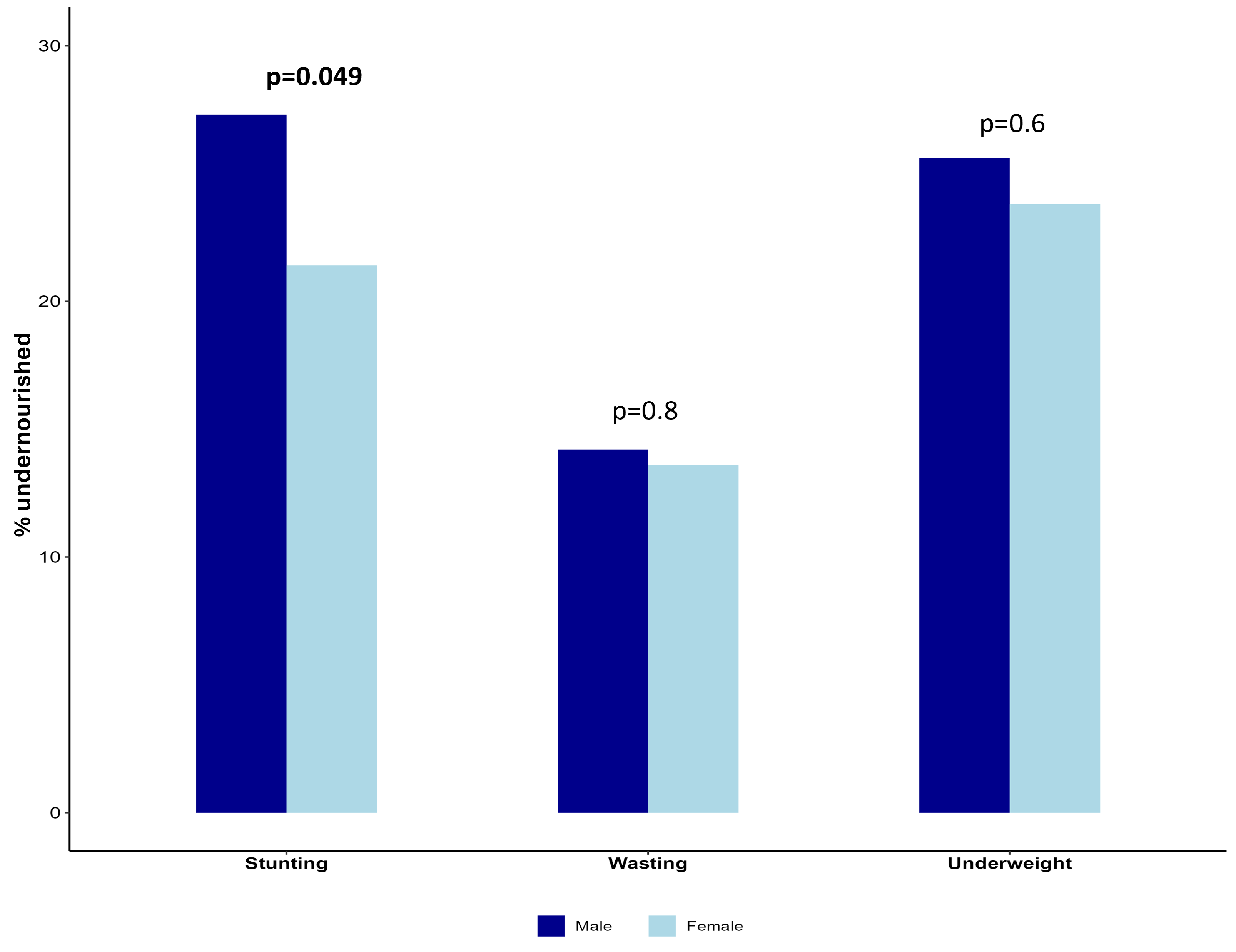

Supplement: Supplementary file 1 — Supplementary Fig. 1: Prevalence of undernutrition at 12–35 months by sex. The prevalence of stunting, wasting and underweight was higher amongst males as compared to females, and was statistically significant for stunting [file 40795_2024_918_MOESM1_ESM.png]
