# Supplementary material for: Functional integration of services during the antenatal period can potentially improve childhood growth parameters beyond infancy: findings from a post-interventional follow-up study in West Bengal, India
Source: BMC Nutr. 2024 Aug 15;10:112. doi: 10.1186/s40795-024-00918-x (PMC11325605; doi:10.1186/s40795-024-00918-x)
Supplement: Supplementary file 2 — Supplementary Material 2 [file 40795_2024_918_MOESM2_ESM.docx]

Supplementary Table 1. Spectrum of antenatal services provided through the ICDS and RMNCH+A programmes in India

| ***Services provided through the ICDS programme*** |
| --- |
| Supplementary nutrition, including the provision of iron and folic acid supplements† |
| Immunization |
| Health check-ups† |
| Referral services |
| Nutrition and health education |
|  |
| ***Services provided through the RMNCH+A program*** |
| Full antenatal care package† |
| Prevention of parent to child transmission of HIV/AIDS (PPTCT) |
| Counselling and preparation for newborn care, breast feeding, birth preparedness |
| Demand generation for pregnancy care and institutional delivery |

†prioritized for functional integration in the study intervention
